# An audit of the use of psychotropic medications over the course of admission to a specialist dementia ward

**DOI:** 10.1192/bjo.2021.918

**Published:** 2021-06-18

**Authors:** Sukhmeet Singh, Margaret Papworth, Concha Turrion, Shamim Ruhi

**Affiliations:** 1 NHS Lanarkshire; 2 Cambridgeshire and Peterborough NHS foundation trust

## Abstract

**Aims:**

The aim of this audit project was to establish the practices in prescribing and de-prescribing of psychotropic medications for patients on a specialist dementia ward.

**Background:**

There is a great deal of evidence demonstration high rates of polypharmacy, defined as ≥5 drugs, in older adults in general and in those with dementia more specifically. NICE guidelines recommend a structured assessment of a patient with dementia to exclude other potential causes, e.g. pain or delirium. Psychosocial interventions are recommended as first line. Antipsychotics should only be offered second line who present a risk to themselves or others. These should only be used for the shortest time possible and reassessed at least every 6 weeks.

**Method:**

Data were collected for patients (n = 20) discharged from a specialist dementia ward between September 2018 and March 2019. The unit has 14 beds caring for patients with predominantly severe behavioural and psychological symptoms associated with dementia (BPSD). The team is comprised of doctors, nurses, a clinical psychologist, occupational therapists, physiotherapists and pharmacists who meet twice a week to review patients. Data were coded by drug class and counts of medication on admission, at the midpoint and at discharge were conducted. Antipsychotic and benzodiazepine dosages were converted into haloperidol and diazepam equivalence.

**Result:**

Of the 20 patients, 70% were male and 30% female. 95% of the patient (n = 19) were admitted under the Mental Health Act (1983). 20% were managed on 1 to 1 observations and 80% were on 15 min observations. In general, the results show little change in the overall rate of psychotropic prescribing. The mean number of psychotropic medications prescribed per patient on admission was 2.30, at the mid-point of admission it was 2.30 and at discharge it was 2.05. Mean benzodiazepine dosage in diazepam equivalence reduced between admission and discharge from 3.20 mg to 2.10 mg. Mean haloperidol equivalent dosages increased at the midpoint of admission from 1.11 mg to 2.27 mg before reducing to 0.78 mg at discharge.

**Conclusion:**

The results demonstrate minimal change in the overall average number and composition of drugs prescribed. There are differences in the use of regular antipsychotics and benzodiazepines between admission and discharge which are consistent with NICE guidelines. Patients had a structured assessment with regular medicines reconciliation supervised by the team pharmacist. Therefore, the ward environment did allow for detailed discussions about de-prescribing which may not be the case elsewhere.

